# Similarities in Blood Mononuclear Cell Membrane Phospholipid Profiles during Malignancy

**DOI:** 10.3390/medsci6040105

**Published:** 2018-11-23

**Authors:** Gohar Hakobyan, Hasmik Davtyan, Kristine Harutyunyan, Knarik Alexanyan, Yelizaveta Amirkhanyan, Anna L. Gharibyan, Liana Asatryan, Yuri Tadevosyan

**Affiliations:** 1 Laboratory of Regulation of Cellular Activity, Institute of Molecular Biology, National Academy of Sciences, 0014 Yerevan, Armenia; hakgohar@gmail.com (G.H.); dav.hasmik@gmail.com (H.D.); chris.harutyunyan@gmail.com (K.H.); yuri.tadevosyan@gmail.com (Y.T.); 2 Center of Oncology after V. Fanarjyan, Ministry of Health RA, 0052 Yerevan, Armenia; knaralex@gmail.com; 3 Center of Hematology after R. Yeolyan, Ministry of Health RA, 0014 Yerevan, Armenia; amirxanyan41@inbox.ru; 4 Department of Medical Biochemistry and Biophysics, Umeå University, SE-901 87 Umeå, Sweden; 5 School of Pharmacy, University of Southern California, Los Angeles, CA 90033, USA

**Keywords:** cancers, mononuclear cells, plasma membrane, phospholipids, biomarkers

## Abstract

Phospholipids (PLs), key elements of cellular membranes, are regulated reciprocally with membrane proteins and can act as sensors for alterations in physiological or pathological states of cells including initiation and development of cancer. On the other hand, peripheral blood mononuclear cells (MNCs) play an important role in antitumor immune response by reacting to cancerous modifications in distant organs. In the current study, we tested the hypothesis that tumor initiation and development are reflected in the alteration pattern of the MNC PL component. We analyzed MNC membrane PL fractions in samples from healthy individuals and from patients with diverse types of cancers to reveal possible alterations induced by malignancy. Compared to healthy controls, the cancer samples demonstrated shifts in several membrane PL profiles. In particular, when analyzing cancer data pooled together, there were significantly higher levels in lysophosphatidylcholine, phosphatidylcholine, and phosphatidylethanolamine fractions, and significantly lower quantities in phosphatidylinositol, phosphatidylserine, and phosphatidic acid fractions in cancer samples compared to controls. The levels of sphingomyelins and diphosphatidylglycerols were relatively unaffected. Most of the differences in PLs were sustained during the analysis of individual cancers such as breast cancer and chronic lymphocytic leukemia. Our findings suggest the presence of a common pattern of changes in MNC PLs during malignancy.

## 1. Introduction

One of the most important factors affecting cancer patient outcome is the time at which cancer is detected, both at initial diagnosis and during tumor recurrence [1]. Currently, the majority of cancers are detected at relatively late stages, leading to ineffective treatment and high mortality rates. The detection of common cancers relies heavily on the imaging modalities, such as computer tomography scans for lung cancer, mammograms for breast cancer, and pelvic ultrasounds for ovarian cancer [2,3,4]. Unfortunately, imaging technologies that allow detection of small lesions indicative of early stages of cancer also frequently result in false positive findings [5,6]. Therefore, establishing novel non-invasive biomarkers to detect cancer at the early stages is of paramount importance for future cancer care, including individualized diagnostics, tumor classification and treatment selection.

Identifying cancer-specific cellular and molecular elements (proteins, mRNAs, circulating tumor cells, metabolites, etc.) in peripheral blood is a highly attractive strategy and can provide affordable screening programs. However, this blood-based approach often suffers from sensitivity and specificity issues due to low concentrations of biomarkers in the blood [7] and rapid in vivo and ex vivo biomarker degradation [8], as well as tumor heterogeneity and highly variable background expression of biomarkers in nonmalignant tissues [9]. Consequently, blood-based diagnostic tests for the cancer detection are scarce [10,11], amplifying the urgent need for new blood-based molecular tests.

Plasma membrane lipids are key elements in numerous processes and are regulated reciprocally with membrane proteins [12]. More specifically, phospholipids (PLs) act as sensors to control alterations in the physiological and/or pathological states of cells. Importantly, alterations in membrane lipids are associated either with adaptation responses or with the etiology of the disease [13,14]. As such, quick and reversible modifications of membrane PL levels and functions are strongly associated with the initiation and development of cancer [15,16,17]. Additionally, some cancer cells acquire resistance to chemotherapy through the changes in their membrane lipid composition [13,18]. Lipidomic studies have also demonstrated that altered PL levels in tissue and blood plasma may be associated with cancer risk, presence, and progression [19,20,21,22].

Patients with different forms of cancer exhibit a poorly functioning immune system. The emergence of a tumor results from the disruption of cell growth regulation as well as from failure of the host to provoke a sufficient immunological antitumor response that is revealed in most cancer patients [23]. Peripheral blood mononuclear cells (MNCs) are comprised of lymphocytes, including T and B cells, natural killer (NK) cells, monocytes, macrophages, and dendritic cells, and play an important role in antitumor responses as well as in the processes of resistance to tumor. As an easily accessible cellular pool of the blood, researchers have focused on MNCs to study the mechanisms for cancer development and for biomarker discovery. A recent in-depth gel-free proteomics approach was used to identify the MNC proteome and set MNCs as a platform for discovery of biomarkers [24]. Despite the advances in this direction, most of the effort has focused on the proteins/peptides as biomarkers for cancer diagnosis and treatment.

Considering the importance of alterations in the plasma membrane profile of lipids, their modification and signal transduction mechanisms in various cell populations of healthy subjects and cancer patients, we hypothesized that cancer initiation and development may be reflected in the overall alteration pattern of lipid characteristics in MNCs plasma membranes. To support this hypothesis, we studied MNC PL profiles in samples from patients with different types of cancers and healthy controls.

## 2. Materials and Methods

*Materials:* Media (Hank’s Balanced Salt Solution (HBSS), Eagle’s Minimum Essential Medium (EMEM)) and reagents for cell isolation from peripheral blood and culture were obtained from Sigma Aldrich Co. (St. Louis, MO, USA). Ficoll-400 was obtained from Serva (Feinbiochemica, Heidelberg, Germany), and polystyrene latex microspheres (2.63%; solid-latex) were obtained from Polysciences (Warrington, PA, USA). Chromatographically pure PL standards were purchased from Avanti Polar Lipids (Alabaster, AL, USA), and thin layer chromatography (TLC) plates (silica Gel G60) were from Merck (Darmstadt, Germany).

*Patients:* All healthy individuals and cancer patients provided informed written consent prior to sampling, according to the Declaration of Helsinki. The present study using human samples was approved by the Ethical Committee (IRB IORG0003427) of the Institute of Molecular Biology of the National Academy of Sciences of Armenia. Studies were carried out on the MNCs isolated from freshly collected peripheral blood sampled from newly diagnosed patients (total of 27) with breast (BrC, *n* = 10), cervical (CC, *n* = 3), testicular (TC, *n* = 1), ovarian (OvC, *n* = 1), prostate (PrC, *n* = 2), and bladder (BlC, *n* = 2) cancers, and chronic lymphocytic leukemia (CLL, *n* = 8), before any treatment. Subjects were aged 35–70 years. Clinical diagnosis was confirmed by a histopathological and cytological examination in the National Center of Oncology after V. Fanarjyan, and National Center of Hematology after R. Yeolyan, MH of Armenia. All patients with solid tumors were evaluated in terms of the tumor–nodes–metastasis (TNM) classification and all of them were diagnosed as non-metastatic (M0). Simultaneously, all the solid tumors were graded at WHO-grade (World Health Organization tumor grading system) ≤2. In addition, patients with hematological malignancies were diagnosed as B (B-cell) CLL with different variants, including typical B-CLL, mixed CLL, and prolymphocytic leukemia. Controls (*n* = 8) were 28–41-year-old healthy individuals without clinical symptoms.

*Isolation of Mononuclear Cells:* Cells were isolated from the heparinized whole blood by standard Ficoll-Hypaque density gradient centrifugation described by Innes et al. [25]. Briefly, blood samples collected in BD Vacutainer CPT (Mononuclear Cell Preparation Tubes) (BD, Franklin Lakes, NJ, USA) with heparin were diluted in HBSS (pH 7.4) and centrifuged at 1500 *g* for 30 min at room temperature on a Ficoll-Hypaque density gradient. Then the MNCs were harvested and washed with the same medium by centrifugation at 3000 *g* for 15 min at room temperature. After resuspension in EMEM (pH 7.4), MNCs were adjusted to a final concentration of 10^8^ cell/mL and kept on ice until membrane isolation. Trypan blue exclusion revealed more than 90% viable cells.

*Subcellular fractionation of cells:* In order to fractionate the cells and obtain plasma membranes, MNCs were disrupted by hypotonic shock and homogenization. Briefly, cells were suspended in ice-cold hypotonic buffer (42 mM KCl, 10 mM HEPES, 5 mM MgCl_2_, pH 7.4) and incubated on ice for 15 min. Cells were sheared by passing them five times through a 30-gauge needle mounted on a 1-mL syringe. The lysates were centrifuged at 500 *g* for 10 min at 4 °C to remove nuclei and cell debris. The post-nuclear supernatant was subjected to centrifugation at 4000 *g* for 15 min followed by 20,000 *g* for 30 min. The resulting pellet containing plasma membranes was resuspended in 50 mM Tris-HCl buffer (pH 7.4). The protein concentration was determined by the Lowry method [26], using the LKB Biochrom 4050 Ultrospec II UV/Vis Spectrophotometer (LKB Biochrom, Cambridge, UK). 

*Lipid analysis:* Lipids from the aliquots of plasma membrane fraction were extracted according to Bligh and Dyer standard method [27]. After this, 1-mL aliquots of chloroform and 0.1 M KOH were added to the chloroform/methanol extracts resulting in phase separation. The lower chloroform phases were washed 2–3 times with distilled water and dried under nitrogen. The obtained residues were dissolved in minimal volume of chloroform, and applied on the silica gel 60 G TLC plates, following PL fractionation by one-dimensional TLC in the solvent system composed of chloroform-acetone–methanol–acetic acid–water (6:8:2:2:1, *v*/*v*). Lipid fractions were visualized by exposing TLC plates to iodine vapors and identified by comparison with chromatographically pure standards.

*Quantification of membrane PLs:* The membrane PL content was quantified by the method of Ernster et al. [28]. Briefly, inorganic phosphorous (P_i_) in various PL fractions was determined after the elution from the TLC plates and incineration in the Kjeldahl flasks with the mixture of HClO_4_ and ammonium molybdate (1%). The color reaction with ammonium molybdate (2.5%) and ascorbic acid (10%) was used and PLs were quantified by elemental phosphorous measurement. The quantities of separate PLs were expressed as μgP_i_/mg of protein.

*Data analysis:* The PL fractions from blood samples of each individual were analyzed in technical duplicates or triplicates with the sampling error averaged in the range of 2.5–18%. Mean values were taken for further statistical analysis. The data from all patients with different types of cancers were pooled in one group and analyzed against control group using Mann–Whitney test. The data are presented in scatter plots with medians and interquartile ranges (IQRs). The significance of differences between the two groups are presented by 95% confidence intervals (CI) and considered statistically significant when the intervals are not overlapping.

## 3. Results

### 3.1. Phospholipid Levels of Mononuclear Cell Membranes in Diverse Cancer Patients vs. Healthy Individuals

**Figure 1:**
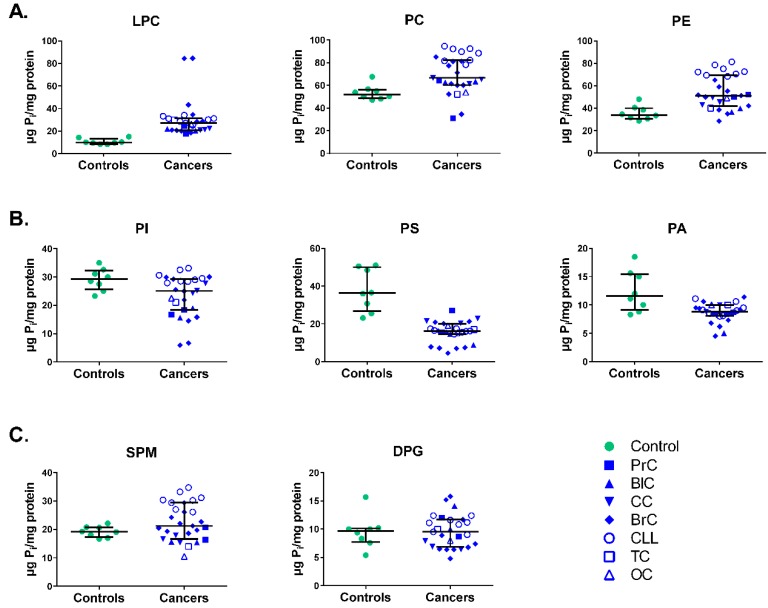
Levels (μg Pi/mg protein) of different phospholipids (PL) fractions in membranes of mononuclear cells (MNCs) from healthy individuals (controls) and cancer patients from seven different cancers grouped together (cancers). (**A**) Lysophosphatidylcholine (LPC), phosphatidylcholine (PC), and phosphatidylethanolamine (PE) fractions demonstrating higher levels in cancers; (**B**) Phosphatidylinositol (PI), phosphatidylserine (PS), and phosphatidic acid (PA) fractions with lower levels in cancers; (**C**) Sphingomyelin (SPM) and diphosphatidylglycerol (DPG) did not show any significant differences between controls and cancers. Data from diverse forms of cancers were pooled together; *n* = 8 for controls; *n* = 27 for cancers. Statistical differences were assessed using Mann–Whitney test and significance assessed with 95% confidence interval (CI).

We analyzed the absolute quantities (μPi/mg protein) for PL fractions from MNC membranes obtained from healthy individuals (denoted as controls) and patients with seven different cancers (denoted as cancers). Eight PL fractions were identified, namely lysophosphatidylcholine (LPC), sphingomyelin (SPM), phosphatidylcholine (PC), phosphatidylinositol (PI), phosphatidylserine (PS), phosphatidylethanolamine (PE), phosphatidic acid (PA), and diphosphatidylglycerol (DPG). First, we pooled data from all cancers and compared to the controls. As illustrated in Figure 1, we found pronounced differences between cancers and controls in several PL fractions. The amounts for LPC, PC, and PE were significantly higher in cancers compared to the controls (Figure 1A). 

Importantly, there was a dramatic shift in the amount of LPC, which was about 3-fold higher during malignancies, i.e., 27 (20.9–31.2) with 95% confidence interval, CI (24.11–37.26) in the cancer group vs. 9.8 (8.6–13.3) with 95% CI (8.5–12.8) in controls. We found smaller but significant differences for PC; 66.5 (61.0–82.2) with 95% CI (63.1–76.4) in cancers vs. 52.05 (48.5–56.2) with 95% CI (48.0–59.0) in controls; and PE 51.3 (42.1–69.4) with 95% CI (48.6–60.3) vs. 34.0 (30.8–40.0) with 95% CI (30.5–41.3) in controls. 

In contrast to the higher levels in LPC, PC and PE fractions, the amounts for PI, PS and PA were significantly lower in MNC plasma membranes from cancer patients (Figure 1B). Notably, there was a more than 2-fold decrease in cancers vs. controls for the PS fraction, with medians of 16.2 ((14.6–20.1), 95% CI (13.5–17.9)) and 36.3 ((26.8–50.0), 95% CI (28.4–47.1)), respectively. In addition, there were small but statistically significant differences for PI and PA fractions (respectively 25.1 (18.4–29.2), 95% CI (20.6–25.4) in cancers vs. 29.3 (25.6–32.2), 95% CI (25.9–32.4) in controls and 8.8 (8.1–10), 95% CI (8.0–9.4) vs. 11.6 (9.1–15.5), 95% CI (9.4–15.4) in cancers vs. controls).

There were no significant differences found for two other PL fractions, i.e., SPM with median 21.2 (16.6–29.4), 95% CI (20.2–25.8) in cancers vs. 19.1 (17.3–20.8), 95% CI (17.6–20.8) in controls; and DPG with 9.5 (6.9–11.7), 95% CI (8.6–10.8) vs. 9.7 (7.8–10.2), 95% CI (7.1–12.1) in cancers vs. controls, respectively.

**Figure 2:**
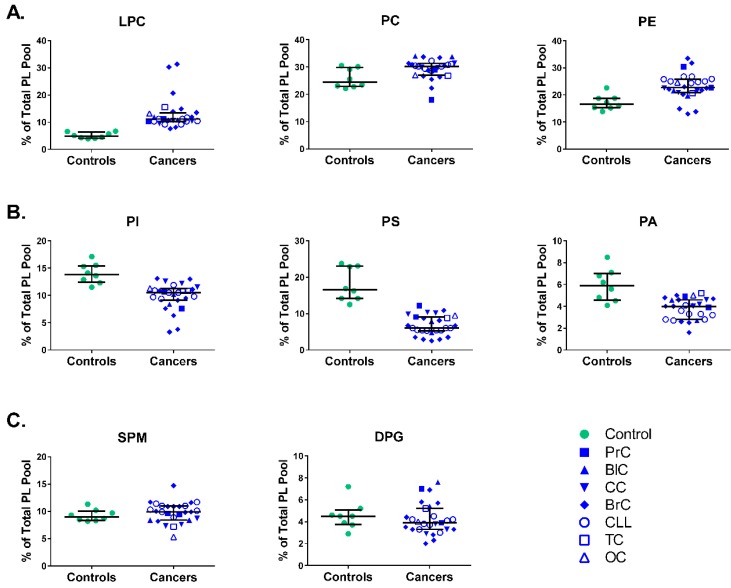
Different PL fractions in membranes of MNCs from healthy individuals (controls) and cancer patients (cancers) presented as % of the total PL pool calculated from the PL pool of each healthy individual or cancer patient. (**A**) LPC, PC, and PE fractions demonstrating higher levels in cancers; (**B**) PI, PS, and PA fractions with lower levels in cancers; (**C**) SPM and DPG did not show any significant differences between controls and cancers. Analysis demonstrated less spread for several PL fractions, i.e., LPL, PC, PE, PI, SPM. Data from diverse forms of cancers were pooled together, see Figure 1. Statistical differences were assessed using Mann–Whitney test and significance assessed with 95% CI.

To determine whether the differences between control and cancer patient data were due to differences in the total PL pools, we calculated the total amount of all the PLs as well as the % content of each PL fraction from the total PL pool calculated for each healthy individual or patient. PL pools for controls and cancers were similar, at respectively 214.8 (189.4–220.7), 95% CI (193.7–222.2) and 229.8 (201.7–273.2), 95% CI (219.2–247.5) μg Pi/mg protein. Based on this, as expected the data on the % content of each PL fraction paralleled the absolute quantity results regarding the direction of the differences (Figure 2). As in the case of the absolute quantities, there were higher proportions for LPC, PC and PE fractions (Figure 2A), lower proportions for PI, PS and PA (Figure 2B) and no differences for SPM and DPG fractions (Figure 2C). The differences, while statistically significant, were within the range of 5–15% for most of the PL fractions.

### 3.2. Phospholipid Content of Mononuclear Cell Membranes in Individual Cancer Patients vs. Healthy Individuals

**Figure 3:**
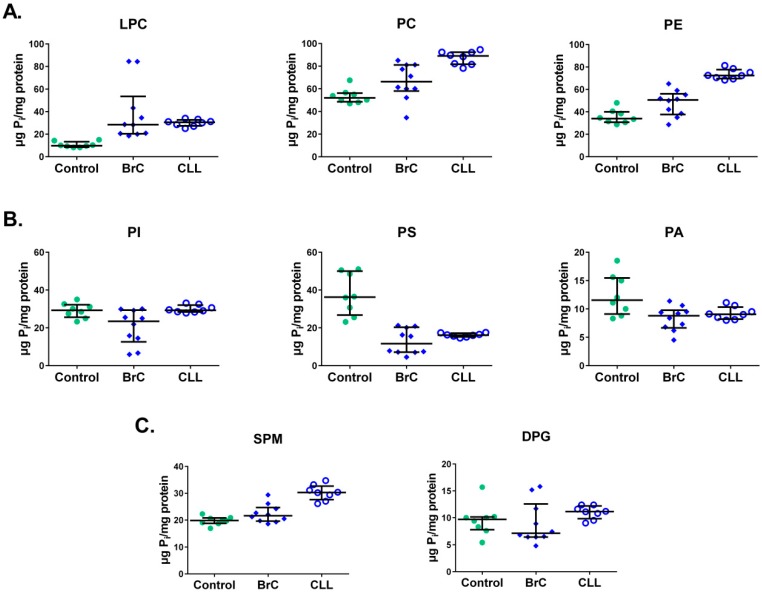
Different PL fraction levels (μg Pi/mg protein) in membranes of MNCs of healthy individuals (control) and from patients with BrC and CLL. (**A**) LPL, PC, and PE; (**B**) PI, PS, and PA; (**C**) SPM and DPG. Most of the differences in PL fractions between individual cancer conditions and controls mostly agree with findings of the PL pool (Figure 1 and Figure 2). Statistical differences between BrC (*n* = 10) or CLL (*n* = 8) and controls (*n* = 8) were assessed using Mann–Whitney test with 95% CI.

Next, we analyzed data to reveal differences in PL levels during separate cancer conditions. Thus, we compared the absolute PL amounts for individual cancer types to the controls. The sample size was sufficient to assume statistical power for only two types of malignancies, breast cancer (BrC, *n* = 10) and chronic lymphocytic leukemia (CLL, *n* = 8). In samples from BrC and CLL, most of the differences in the amounts of PL fractions were in agreement with the findings of all the cancer data pooled together. There were higher levels of LPC, PC and PE fractions compared to controls (Figure 3A). As in pooled samples, the analysis of BrC samples demonstrated lower levels for PI, PS and PA fractions (Figure 3B) and no significant difference in the SPM levels (Figure 3C).

In contrast, in CLL samples there was no difference in the levels of PI (Figure 3B), and SPM level showed statistically significant increase compared to controls (Figure 3C). In addition, similar to the findings with pooled data, there were no differences for DPG levels in both BrC and CLL compared to controls (Figure 3C).

## 4. Discussion

Early detection of cancer is of paramount importance for diagnosis and effective treatment strategies that can result in increased survival rates of cancer patients. Despite substantial advances in this field, the development of early cancer detection biomarkers with a predictive value is still very challenging. In particular, lipids are still overlooked as potential biomarkers for cancer despite the ample evidence that malignancies affect lipid composition and metabolism. Moreover, the studies have been focusing on changes occurring within the cancerous tissues and cells [29,30,31]. However, mounting evidence suggests that circulating immune cells can respond to the cancerous modification in distant organs systems [32,33]. Therefore, in this study we analyzed the PL component of MNCs from the peripheral blood of healthy individuals and patients with diverse cancers to reveal any common patterns during different cancer conditions.

We found substantial differences in several membrane PL profiles of MNCs obtained from different cancer patients with different types of malignancy compared to healthy individuals. In particular, analysis of pooled data from all cancer samples demonstrated significantly higher levels in the absolute amounts of three different PL fractions, namely LPC, PC, and PE, in comparison to the levels in membranes of healthy individuals. Furthermore, three PL fractions such as PI, PS, and PA were significantly lower, whereas two other PL fractions, SPM and DPG, were not significantly different between healthy individuals and cancer patients when data were pooled together. These differences, albeit with lower extent, were sustained when analyzed as a percentage of each fraction from the total PL pool. Moreover, most of the differences in the MNC membrane PL content were reproduced for individual cancers. In this regard, the differences in PL fractions were completely reproduced in BrC samples. In the CLL samples, the differences were in agreement with those found for the analysis of pooled samples except for two PL fractions, PI and SPM. Compared to the controls, levels of PI were not different, and the SPM level was significantly higher. Observed differences between BrC and CLL samples may be due to the type of the cancers; solid tumors as in case of BrC vs. hematological cancers represented by CLL.

Despite altered individual PL profile, we did not find differences in the MNC total PL content between healthy and cancer groups. Evidence suggests there is enhanced lipid synthesis in cancer [34] to satisfy the demand in structural and functional cellular components. This can result in enhanced membrane area [35], increased activity of fatty acid synthase, and lipid accumulation within the cells as lipid droplets [36,37]. These changes are more characteristic for the cancer cells themselves and most probably do not reflect on the MNC PL content. Nonetheless, significant differences in the absolute levels of individual MNC PL fractions revealed in our study were also present when calculated as percentage from the total PL pool. Interestingly, in case of percentage distribution of the individual PL fractions the differences between controls and cancers were within 5–15%. This observation suggests that even under pathological conditions the PL proportions within plasma membrane are tightly regulated, most probably, for maintaining the integrity of cellular membranes.

Among the PL fractions, LPC fraction showed the most pronounced difference between cancer patients and healthy individuals (2–3 times higher levels in absolute values). This finding is in agreement with previously published works demonstrating that LPC fraction is significantly alerted in diverse cancer cells and tissues or in blood plasma [19,20,21,22]. Increased LPC levels lead to increased membrane disordering/fluidity, a characteristic of cellular membranes during cancer [20,35,38]. We also found higher levels in the PC and PE fractions in the cancer pool. These differences were reproduced in individual cancers with statistical significance reaching in BrC and CLL for respectively PC and PE levels. The previously published findings on the changes in PC and PE fractions are very diverse and sometimes controversial [39,40,41]. For example, substantial evidence suggests that PC levels are elevated during cancers, whereas significant decrease was noted in breast cancer cell lines [42].

All the PL pools at the steady state level are a result of simultaneous synthesis and catabolism as well as PL interconversions, therefore, our findings suggest that the enhanced catabolic process in MNC membranes may partially compensate the de novo synthesis during cancer. For example, higher LPC during cancer may be a result of enhanced activities of phospholipase A2 found during cancers [35,43,44]. This was not largely reflected on the PC level due to its overall high content in the plasma membranes; however, for some of the PL fractions such as PI, PS, and PA, the catabolic processes likely overpowered their formation. As such, dramatic loss of PS fraction during cancer may be a result of activation of catabolic processes involving PS fraction. This may likely be a result of PS decarboxylation leading to greater levels of PE and PC per PL fractional interconversions PS → PE → PC [45,46]. In another scenario, greatly reduced PS levels during cancer may also be due to activation of PS-specific phospholipase A1. In support of this, a recent study found that PS-specific phospholipase A1 expression is associated with tumor invasion and metastasis in colorectal cancer [47]. These assumptions need further in-depth investigation.

Altered lipid content in blood plasma of cancer patients has been demonstrated previously [48]. These levels often vary due to dietary influences [49]. Given the growing evidence of the role of the immune cells in fighting/controlling cancerous growth in the body, we propose a better approach of focusing on the blood MNCs. MNCs represent a pool which contains immune cells that undergo significant changes during malignancies and result in inability to clear/fight cancer cells as well as contributes to the resistance of the cancer cells to therapies. Importantly, the proportion of different cells in the MNC pool also undergoes changes during early stages of cancer as suggested for example by the finding of prevalence of regulatory T-cells (T_regs_) in the blood at initiation and development of variety of cancers [50]. It is plausible that differences in PL fractional content originate from various cell types within the MNC pool, however, the important finding from our studies is the specific pattern of the differences in the major PL fraction levels during malignancy. This suggests that there are largely similar mechanisms involved in PL modification processes in MNC membranes regardless of the cancer type; solid tumor such as BrC or hematological malignancy like CLL.

Some of PL fractions (LPC, PS, and PE), altered in cancer tissues, cells or blood plasma of patients with different cancers, were suggested to be of use as additional markers for cancer risk and detection [19,20,21,22,51,52]. PL metabolic processes such as PL catabolism and PL interconversions are tightly regulated and thus changes may be occurring in several PL fractions simultaneously. Therefore, patterns of several related PL profiles rather than the individual PL levels in MNC membranes may be more representative of abnormal, malignant processes in cancer patients. Our finding on commonality of PL profiles across different cancer types supports this notion. Future studies will address whether simultaneous changes in several PL fractions can result in alterations of lipid microenvironment of proteins/receptors such as checkpoint molecules and/or oncogenes in PMNs leading to immune dysfunction during cancer.

In conclusion, we have demonstrated: (1) significant differences in PL profiles of MNCs between cancer patients and healthy individuals; and (2) common PL profile in different types of cancer. Notably, to further support findings of this initial study, more extensive tests with larger sample size on various cancers and in-depth analyses of PL fractions in terms of their fatty acid composition are needed. These studies will set the stage for future development of MNC-based PL biomarkers for early detection and evaluation of cancer.
